# Cerebellar gangliocytoma as a cause of fluctuating hearing loss

**DOI:** 10.1002/ccr3.3158

**Published:** 2020-09-13

**Authors:** Ana Julia Rocha, Reyes Márquez, Jose Ramon García‐Berrocal

**Affiliations:** ^1^ Servicio de Otorrinolaringología Hospital Universitario Puerta de Hierro Madrid Spain

**Keywords:** cerebellar ganglyocitoma, cerebrospinal fluid pressure, fluctuating hearing loss

## Abstract

Cerebellar lesions could be a cause of fluctuating hearing loss, due to the increase of intracranial pressure by partially or complete blocking of the Foramen of Luschka. Patients with intracranial hypertension may present audio‐vestibular symptoms. Fluctuating sensorineural hearing loss may be the manifestation of different inner ear disorders such as Meniere's disease (MD), immune‐mediated inner ear disease (IMIED), otosyphilis, and labyrinthine fistula including semicircular canal dehiscence. A rare mechanism involved in the fluctuating hearing loss is the increase in cerebrospinal fluid (CSF) pressure, that may be caused by a cerebellar tumor. A 51‐year‐old female presented a 2‐year history of left ear fluctuating hearing loss and tinnitus, with fluctuations among the day, and normal otoscopy. Several audiometries showed a left ear moderate sensorineural hearing loss in low frequencies (Figure 1). The patient underwent a cerebral magnetic resonance imaging (MRI) (Figure 2). She was treated with three courses of systemic steroids showing improvement of symptoms during the treatment. However, the symptoms always returned when corticotherapy was interrupted. The patient was given two intratympanic steroids cycles combined with hydrochlorothiazide/ amiloride hydrochloride. The cerebral MRI described a left cerebellar focal lesion diagnosed as a cerebellar gangliocytoma. After receiving the second intratympanic steroids, cycle combined with systemic ameride showed a significant improvement of audition. Between the several causes of fluctuating hearing loss, a cerebellar gangliocytoma is a very rare disease, which needs a high degree of suspicion and otorhinolaryngologists should be familiar with this entity since patients may present with audiological and vestibular symptoms.

## Graphical abstract

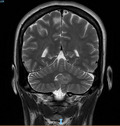

## 1 INTRODUCTION

Fluctuating hearing loss may be defined as a reversible and variable length hearing loss, in the same ear, that usually associates to tinnitus, vertigo, and aural fullness. Some possible causes of fluctuating hearing loss are Meniere's disease, immune‐mediated inner ear disease (IMIED), otosyphilis, and labyrinthine fistula including semicircular canal dehiscence.

## 2 CASE REPORT

**Figure 1:**
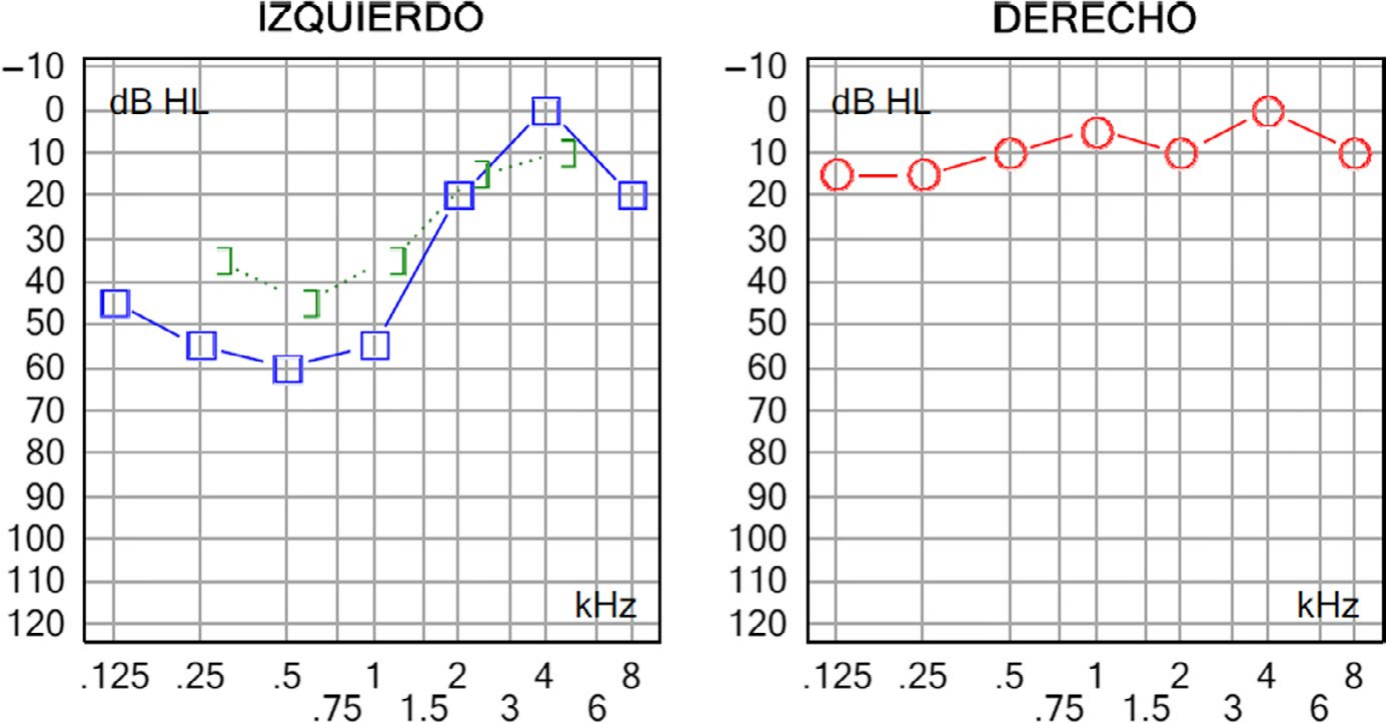
Audiometry show a left ear moderate sensorineural hearing loss in low frequencies

**Figure 2:**
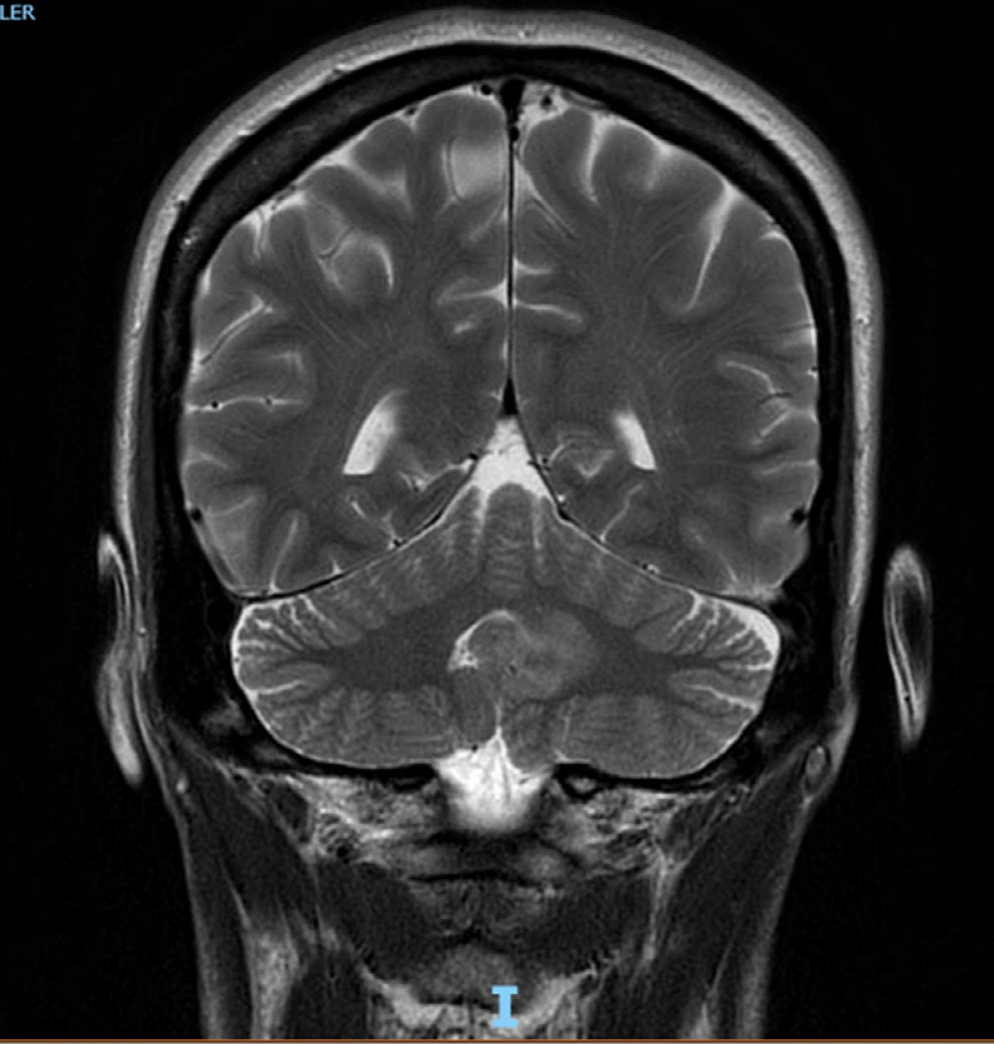
MRI show a hyperintense left cerebellar focal lesion on T2‐weighted imaging

A 51‐year‐old female presented a 2‐year history of left ear fluctuating hearing loss and tinnitus, with fluctuations among the day, without a clear relation to posture, not associated to dizziness or vertigo. Also presents morning headaches that disappeared 2 hours after she wakes up. Physical examination revealed a normal otoscopy. She was treated with three courses of systemic steroids (methylprednisolone: descending dosis starting with 1mg/kilogram/day) showing improvement of symptoms during the treatment. However, the symptoms always returned when corticotherapy was interrupted. The patient was given three intratympanic steroids cycles (0.7 mL methylprednisolone), symptoms improved in the second cycle coinciding with the start of treatment with hydrochlorothiazide/ amiloride hydrochloride. Several audiometries showed a left ear moderate sensorineural hearing loss in low frequencies (Figure 1). The patient underwent a cerebral magnetic resonance imaging (MRI) (Figure 2) that described a hyperintense left cerebellar focal lesion of 19 × 17 × 19 mm on T2‐weighted imaging, arising from the junction of the left cerebellar hemisphere, the lateral left wall of the fourth ventricle, and the cerebella vermis. The lesion partially obliterated the Foramen of Luschka, without signs of obstruction. No enhancement with intravenous contrast or vasogenic edema was described.

A diagnosis of Cerebellar Gangliocytoma was made.

## 3 DISCUSSION

Gangliocytomas are low‐grade tumors of central nervous system, composed by well‐differentiated neurons, that can arise in any location of the neuroaxis, showing predilection for supratentorial locations (temporal lobes).

MRI images show tumors hyperintense on T2, with variable contrast enhancement: none, ring/solid enhancement. These lesions may have cysts (50% of cases) or calcifications (30%) inside.

It is a rare disease with a low incidence, and it is more common in children and young adults. If the tumor required intervention, the main treatment is surgery.

Cerebellar gangliocytoma is part of a group of uncommon tumors constituted by neuronal‐glial tumors, with variable degree of neuronal and glial differentiation. Other tumors classified in this category are as follows: dysembryoplastic neuroepithelial tumor, dysplastic gangliocytoma of the cerebellum (Lhermitte‐Duclos disease), papillary glioneuronal tumor, paragangliomas, among others.

Dysplastic cerebellar gangliocytoma, Lhermitte‐Duclos disease, is an extremely rare tumor that affects cerebellar architecture, with abnormally hypertrophic ganglion cells and hypoplasia of cerebellar white matter. Arises from hemispheres but may extend to vermis. Clinical presentation is characterized by cerebellar dysfunction symptoms and obstructive hydrocephalus. Its typical MRI image is nonenhancing, well‐delimited lesion, T1 hypotense, and laminar pattern in T2 images with alternating high and low signal.

The radiological pattern of this case does not suggest a Lhermitte‐Duclos disease.

A rare mechanism involved in the fluctuating hearing loss is the increase of cerebrospinal fluid (CSF) pressure. It may be transmitted to the subarachnoid space surrounding the acoustic nerve and to the perilymphatic space resulting in arrest of the blood supply to the acoustic nerve and impaired movement of the footplate of the stapes.

Patients affected by idiopathic intracranial hypertension and patients suffering from tumors blocking the CSF flow may present audio‐vestibular symptoms.

In the present report, MRI imaging revealed a tumor (cerebellar gangliocytoma) in close proximity to the left wall of the fourth ventricle, partially blocking the Foramen of Luschka.

Fluctuating hearing loss in the left ear was confirmed in our patient, and a transient intracranial pressure modification was hypothesized as the probable cause.

Several mechanisms have been postulated:

‐The acoustic nerve may suffer functional disturbances or anoxia due to transmission of intracranial pressure through the sheet from the subarachnoid space
‐Direct pressure transmission via the perilymphatic space or the cochlear aqueduct resulting in reduced cochlear blood flow, venous congestion, stretching of the basilar membrane, inducing perilymphatic and endolymphatic pressure alterations, and thereby hindering mobility of the footplate.

Our patient showed lower frequency hearing loss suggesting a similar mechanism as in the endolymphatic hydrops observed in MD and IMIED ^1^.

Hearing loss improved with the administration of systemic and intratympanic corticosteroids since these drugs may induce expression of aquaporin‐1 and increase transcellular water transport in the inner ear, mainly in the stria vascularis and the endolymphatic sac.^2^ A reduced expression of aquaporin‐1 in the choroid plexus epithelium of hydrocephalic rats has been reported.^3^

The symptoms also improved with diuretics, which decreased CSF production and intracranial pressure. This fact also supports the hypothesis of hearing loss induced by transient intracranial pressure modification due to a cerebellar tumor.

In conclusion, cerebellar gangliocytoma is a very rare disease, which needs a high degree of suspicion in patients with a cerebellar mass, and otorhinolaryngologists should be familiar with this entity since patients may present audiological and vestibular symptoms, occasionally fluctuating hearing loss.

## CONFLICT OF INTEREST

None declared.

## AUTHOR CONTRIBUTIONS

Ana Julia Rocha and Reyes Marquez Altemir: have been involved in drafting the manuscript and revising it critically for important intellectual content. Jose Ramón Garcia Berrocal: has given final approval of the version to be published.

## ETHICAL APPROVAL

The patient gave verbal consent for including data and radiological images in this publication.
